# Fatigue, pain interference, and psychiatric morbidity in multiple sclerosis: The role of childhood stress

**DOI:** 10.1371/journal.pone.0292233

**Published:** 2023-10-18

**Authors:** Carri S. Polick, Robert Ploutz-Snyder, Tiffany J. Braley, Cathleen M. Connell, Sarah A. Stoddard

**Affiliations:** 1 School of Nursing, Duke University, Durham, NC, United States of America; 2 Durham VA Health Care System, Durham, NC, United States of America; 3 School of Nursing, University of Michigan, Ann Arbor, MI, United States of America; 4 Division of Multiple Sclerosis & Neuroimmunology, Department of Neurology, Michigan Medicine, Ann Arbor, MI, United States of America; 5 School of Public Health, University of Michigan, Ann Arbor, MI, United States of America; Universita degli Studi di Napoli Federico II, ITALY

## Abstract

**Background:**

Multiple sclerosis (MS) is a progressive, autoimmune disease of the central nervous system that affects nearly one million Americans. Despite the existence of immunomodulatory therapies to slow physical and cognitive disability progression, interventions to ameliorate common symptoms of MS, including fatigue and pain, remain limited. Poor understanding of risk factors for these symptoms may contribute to treatment challenges. In recent years, childhood stress has been investigated as a risk factor for chronic autoimmune conditions including MS; yet remarkably few studies have investigated the relationship between childhood stressors and chronic MS symptoms. Our aim was to examine clusters of stressors and three key features of MS: fatigue, pain interference, and psychiatric morbidity.

**Methods:**

Cross-sectional data were collected from a sample of People with MS (PwMS) via a national web-based survey that assessed the presence and type of childhood stressors and MS clinical features. Hierarchical block regression was used to assess associations among emotional, physical, and environmental childhood stressors and three clinical features commonly experienced by PwMS.

**Results:**

N = 719 adults with MS (aged 21–85) completed the survey. Childhood emotional and physical stressors were significantly associated with overall presence of fatigue (p = 0.02; p<0.03) and pain interference (p<0.001; p<0.001) in adulthood, as well as the magnitude of both outcomes. Environmental stressors (p<0.001), in addition to emotional (p<0.001) and physical (p<0.001) stressors were significantly associated with psychiatric morbidity in PwMS.

**Conclusion:**

Childhood stress may predict fatigue, psychiatric morbidity, and pain in adults with MS. Further research is needed to show cause and effect; however, if an association exists, strategies to mitigate the impact of childhood stress could offer new pathways to reduce the severity of these symptoms. Broadly, this work adds to the body of evidence supporting upstream preventive measures to help address the stress on children and families.

## Background

Childhood adversity is a public health crisis, traditionally encompassing emotional and physical abuse/neglect, sexual abuse, parental mental illness, substance abuse, or separation, and other household dysfunctions such as witnessing violence before the age of 18 [1]. Two thirds of the United States (US) population have experienced these Adverse Childhood Experiences (ACEs), otherwise known as child maltreatment, traumatic, or toxic stressors [2]. There has also been an expansion to include adversities such as poverty, discrimination, community dysfunction, living in foster care, social rejection, and harsh discipline (e.g., spanking), as these stressors similarly produce stress responses [3–8].

Traumatic stressors elicit many physiologic processes, including increased inflammation, reduced coping/resilience, poor health behaviors, and neurodevelopmental problems, contributing directly and indirectly to numerous acute and chronic health issues later in life [7, 9, 10]. A hallmark dose-response relationship exists between cumulative ACEs and worse outcomes, with higher levels of ACEs associated with worse long-term health [1, 9].

Traumatic childhood stressors have been associated with immune-mediated inflammatory diseases (IMID) [11, 12]; however, few studies have investigated relationships between childhood stressors and Multiple Sclerosis (MS)–the most common cause of non-traumatic disability among young adults [13, 14]. Even fewer have focused on specific clinical features associated with MS [15–19]. Multiple Sclerosis is characterized by neuroinflammation and neurodegeneration of the central nervous system that results in both physical and cognitive disability [20–23]. MS impacts nearly one million people in the US, with a female to male ratio of 3:1 that appears to be widening [24]. It is most commonly diagnosed in White women between the ages of 20–40 years old, however, emerging work to better evaluate and support people with MS (PwMS) with varying lived experiences suggests that MS may be more common across diverse groups than previously thought [24–26].

In addition to focal neurologic dysfunction, MS is associated with “invisible symptoms” including fatigue, pain, and mood disturbances, which each carry a high lifetime prevalence in PwMS and confer significant morbidity [27, 28]. At least 50% of PwMS experience depression, which commonly clusters with anxiety [29, 30]. Evidence shows that pain fluctuations can predict both physical and social functioning in PwMS, while fluctuations in depressed mood and fatigue are associated with lower levels of emotional wellbeing [31]. A person-centered approach recognizes that fluctuations of these symptoms from an individual’s baseline–which could be triggered by past events—can impact the daily lives and wellbeing of PwMS [31]. Given limitations in current treatment strategies for specific MS symptoms including fatigue and pain, it is imperative to develop a better understanding of the biopsychosocial basis of these symptoms to shape more personalized pharmacological and non-pharmacological treatments.

Research has only recently started to examine links among traumatic child experiences and fatigue [19] and pain catastrophizing [32] for PwMS or IMID which included PwMS. Childhood adversity has been associated with worse anxiety the year following an MS diagnosis [15] and psychiatric morbidity (e.g., diagnosis or symptoms of anxiety, depression, PTSD) in an IMID sample [33]. While this emerging area of research is compelling, measurement differences regarding stressors (e.g., type, count, severity) and MS covariates makes comparisons difficult. Medication (e.g., analgesics, antidepressants) and Disease Modifying Therapies (DMTs) impact the disease course, lives, and wellness of PwMS, yet have not always been fully accounted for in previous studies [14]. Further, the MS sample compositions in these few studies have generally been limited in size (e.g., n = 31, n = 232, n = 571) and/or limited to one geographic location [14, 15, 19, 32, 33]. Most PwMS (60–80%) experience hot or cold temperature intolerance which can worsen sensory symptoms [34]. Thus, studies with wider geographical diversity are less susceptible to the effects of extreme weather in any one location and can help provide more robust symptom evidence.

Studies that substantiate previous findings, build upon prior data with different measurement strategies, and examine additional MS clinical features are sorely needed. As opposed to measuring cumulative count or individual ACEs, a novel way to measure stressors includes clustering by impact level. For example, stressors that happen *to* a child may impact them differently than stressors that happen *around* them–in their environment. Stressors that happen directly to a child may also yield a different effect based on whether the child was physically harmed or not. Therefore, clustering stressors as emotional, physical, and environmental may help determine which types are most detrimental.

The Social Safety Theory proposes that real or perceived threats to an individual’s safety can elicit the stress response to alter physiological processes (e.g., inflammatory response), perception of future threats, and responding behavior (e.g., social, emotional, cognitive) [7]. We propose that traumatic childhood stressors are threats to social safety which could alter the lives of PwMS in ways that impact their disease through inflammatory, cognitive, or behavioral pathways. The aim of this study was to examine the associations between child stressors and three common symptoms experienced by PwMS: fatigue, pain interference, and psychiatric morbidity. We hypothesized that childhood emotional stressors, childhood physical stressors, and childhood environmental dysfunction would each uniquely contribute to MS symptoms.

## Materials and methods

Approval was obtained from the University of Michigan IRB. Participants read informed consent materials during the screening process and agreed to an implied consent statement to proceed with the study. Following the approach from previous studies, we collected data from US-based adults diagnosed with MS through a national, online self-report survey deployed through the National MS Society (NMSS) [35, 36]. Participants were primarily recruited by email from the NMSS listserv of PwMS who are interested in research opportunities; a survey link was also posted on the NMSS website and social media. The listserv contains about 80,000 emails, while the number of PwMS in the US is nearly one million [37]. Previous studies using this listserv have shown that only about half opened the email, and that most respondents are female (80%), white (91%-98%), with relapsing-remitting MS (67%-69%)[35, 36]. Recruitment lasted from September 15th to November 4^th^, 2021. STROBE guidelines were used to strengthen the reporting and transparency of this observational study [38].

### Measures

Demographic and MS data were collected, including age, MS subtype (e.g., relapsing-remitting [RRMS], primary or secondary progressive [PPMS, SPMS]). Medications including analgesics, antidepressants, and anxiolytics that could impact the pain interference and psychiatric outcomes were accounted for by using a count of mediation classes (0–5). Disease Modifying Therapies (DMTs) were accounted for in the pain interference and fatigue outcomes. The demographic and MS variables were used as baseline covariates (Table 1).

**Table 1:** Hierarchical block modeling approach.

	Predictors in each block model
Base model 1: demographic and MS covariates	Age, gender, education, MS type, *pharmacological treatment tailored per outcome and listed in outcome tables 4–6
Model 2 adds emotional block	Base model + emotional abuse severity & duration
Model 3 adds physical block	Model 2 + physical abuse severity & duration, sexual abuse severity & duration, harsh discipline severity & duration
Model 4 adds environmental block	Model 3 + cumulative count of environmental stressors

### Predictor variables

Childhood stressors were measured with the Stress and Adversity Inventory (STRAIN) which assesses stressors across the lifespan, and was validated using the Childhood Trauma Questionnaire [39]. The STRAIN captures up to 455 variables which can then be applied per the research question [40]. As mentioned in the introduction, the focus of this paper was to cluster child stressors by potential impact level, using childhood stressors that align closest with traditional and expanded ACE items. Many expanded ACEs are now recognized as Social Determinants of Health such as neighborhood safety or feelings of exclusion/discrimination based on personal factors [41]. Participants were asked about whether a stressor happened and if so, follow up questions captured the age at which a stressor started, stressor severity from not at all (0) to extremely stressful (5), and the duration (years/months). Variables were classified as childhood stressors if a stressor started before the age of 18, and durations were not limited. Thus, it was possible for the duration to extend beyond age 18, however, this aligns with how count-based measures categorize stressors and allows better comparison between the two approaches. Severity and duration were each summed per type of stressors. *Childhood emotional stressors* included indicators of emotional abuse (i.e., severity, duration; Table 1). *Childhood physical stressors* included the severity and duration of physical abuse, recurring sexual abuse, and harsh discipline (i.e., spanked, hit, or hurt). To be comprehensive and include a wider stressor experience, yet parsimonious for analytic modeling, *childhood environmental dysfunction* was a count variable, aligning with traditional ACE methods. Nine variables were summed into one count to capture the exposure to parental mental illness and/or substance use, fighting, witnessing abuse, child being separated from the parent, parental divorce, housing instability, feeling unsafe in their neighborhood, feeling excluded at work based on factors like race or gender, and having their home broken into.

#### Outcome variables

The concept of psychiatric morbidity has been measured in many ways with different tools throughout the literature, generally encompassing elements of psychiatric diagnoses and symptom severity [33, 42–45]. Similarly, for our study we define psychiatric morbidity as the cumulative burden of diagnoses and symptoms, to capture PwMS who may have been diagnosed yet are asymptomatic, as well as PwMS who may be symptomatic but undiagnosed. Additionally, we expand beyond this to Include events like emergent hospitalization which helps represent symptom burden with more objective data, as hospitalization likely would not occur unless a healthcare provider found it necessary. Therefore, we measured 1) anxiety diagnosis, 2) depression diagnosis, 3) Posttraumatic-stress disorder or other psychiatric diagnosis (e.g., schizophrenia), 4) symptomatic anxiety, 4) symptomatic depression, 5) emergency hospitalizations for psychiatric or substance use issues. Symptomatic anxiety and depression were measured with Patient Reported Outcomes Measurement Information System (PROMIS) scales. The PROMIS system is a publicly available repository of valid self-report measurement instruments funded by the National Institutes of Health (NIH) [46]. Four-item short forms were used and responses were dichotomized as symptomatic or asymptomatic if participants reported any symptoms or no symptoms within the last week. The remaining diagnoses and events were self-report binary questions. To be inclusive yet parsimonious regarding analytic modeling, dichotomized scores were summed with a possible range of 0–6 with higher scores representing more psychiatric morbidity.

Fatigue and pain interference were measured with PROMIS scales. Raw scores were converted to standardized T-scores to compare results to a healthy general population, with a mean of 50 and standard deviation of 10 [47]. The PROMIS-Fatigue MS Short Form is an 8-item scale measuring fatigue along with its emotional impact and impact on daily living over the last week for PwMS [46, 48]. Items are scored on a Likert scale ranging from (1) never to (5) always, with higher scores representing higher fatigue. Internal consistency for this study was very high (Cronbach’s alpha 0.95)

The PROMIS-Pain Interference 8a is an 8-item scale measuring the magnitude that pain had interfered with mental, physical, and social aspects of an individual’s life over the last week and has previously been used in the MS population [35, 46, 47, 49]. The items are scored on a Likert scale from (1) not at all to (5) very much, with higher scores representing more pain interference. Internal consistency for this study was very high (Cronbach’s alpha 0.99)

### Sample size determination

We conducted a power analysis to determine the necessary sample size to have 80% power to detect significant changes in model fit between nested models. A minimum sample size of n = 332 was needed to detect a 4% improvement, assuming a 2-tailed alpha (0.05). These analyses employed case-wise deletion, so only participants with complete data per each outcome were included in the final samples.

### Analyses

Broadly, our approach uses blocks of similar predictors to build sequentially more complex models upon a base model to assess the contributions to latent constructs of stressors and model fit. If a block of predictors was found to not contribute, it was removed from the final model, but not the analyses altogether. This means that the same sample was used in both analyses, with and without that block, and that removal of the block did not allow for more participants to enter the analyses. This allowed for better model fit comparisons. Once a final model was selected, individual predictors were assessed. Table 1 displays the hierarchical modeling and which predictors are in each block. Assumption testing (e.g., normality, heteroskedasticity) was completed and used to determine more specific analytic strategies.

Specifically, our approach was to determine if childhood stressors grouped by type (e.g., emotional, physical, or environmental) contribute to each of the three outcomes. As seen in Table 1, the base model consisted of demographic and MS specific covariates relevant to each outcome, to assess baseline contributions. We then added the childhood emotional stressor block as seen in model 2 and compared 1) model fit using Akaike Information Criterion (AIC), 2) significant contributions over and above what the base model contributed with likelihood ratio testing, 3) variance contributions of R^2^ and pseudo R^2^. This process was repeated each time for adding the childhood physical stressor block in model 3 and the childhood environmental stressor block in model 4. It is important to note that this strategy of clustering similar stressors assumes some collinearity, to represent latent constructs of the types of stressors, and thus may underestimate individual relationship shown in outcome tables.

Poisson regression was used for the count of psychiatric morbidity. Two-part modeling was used for the two PROMIS scale outcomes of fatigue and pain interference due to a mixed distribution. T-scores that represented “no pain” and “no fatigue” were replaced with zeros. Two-part modeling first used logistic regression to analyze yes/no for having *any* fatigue or pain interference. Contingent on having any fatigue or pain interference, the second part used OLS linear regression to analyze the *magnitude* of the fatigue or interference.

## Results

### Participants

The final sample, encompassing participants who informed any of the final models [50], consisted of 719 PwMS. Consistent with previous research using NMSS listserv and other large MS samples [35, 37, 51], the majority were female (84%), White (88%), with Relapsing Remitting MS (79%), were on average 31(SD 10) years old at symptom onset, and had at least a bachelor’s degree (Table 2). Compared to a general population with a standardized T-score of 50, this sample had higher mean fatigue (56) and pain interference (53). The most prevalent childhood stressor type was harsh discipline (n = 366, 50.9%), followed by emotional abuse (n = 240, 33.4%), physical abuse (n = 114, 16.7%), and sexual abuse (n = 92, 12.8%), with mean duration times spanning 60–190 months overall (Table 3).

**Table 2:** Sample characteristics.

**Age** M(SD) (n = 719)	49.3(12.7) range: 21–85
**Gender** n(%) (n = 719)	
Female	602 (84%)
Male	102 (14%)
Transgender, non-binary, gender non-conforming or other	15 (2%)
**Race / Ethnicity** n(%) (n = 477)	
White	421 (88%)
Black	23 (5%)
Latinx	2 (<1%)
Asian	4 (<1%)
American Indian or Alaska Native	2 (<1%)
Native Hawaiian or Pacific Islander	1 (<1%)
Bi-racial or mixed	24 (5%)
**Education** n(%) (n = 719)	
High school, GED, or below	36 (5%)
Associates degree or some college	169 (24%)
Bachelor’s degree	260 (36%)
Master’s degree or above	254 (35%)
**Smoking status** n(%) (n = 715)	
Never smoker	468 (65%)
Former smoker	200 (28%)
Current or social smoker	47 (7%)
**MS Subtype** n(%) (n = 719)	
RRMS	565 (79%)
PPMS	35 (5%)
SPMS	87 (12%)
PRMS	9 (1%)
Unsure	23 (3%)
**Length of time since MS onset** M(SD) (n = 719)	18.5 (12.3) range: 0–59
**Disease Modifying Therapy (DMT)** n(%) (n = 715)	
None	129 (18%)
First line	262 (39%)
Second line	310 (43%)
**Count of medication classes** that can impact pain M(SD) (n = 705)	1.64 (1.3) range: 0–5
**Disability (PDDS)** n(%) (n = 712)	
Mild	368 (52%)
Moderate	238 (33%)
Severe	106 (15%)
**Age at MS symptom onset** M(SD) (n = 709)	31 (10)
**Outcome variables**	
**Fatigue,** median (IQR), M(SD) (n = 719)	58 (52–63), 56(13)
**Pain interference,** median (IQR), M(SD) (n = 719)	54 (0–62), 53(10)
**Psychiatric morbidity count** M(SD) (n = 719)	2.2 (1.7) range: 0–6

**Table 3:** Stressor characteristics.

Severity	Emotional abuse (n = 719)	Physical abuse (n = 719)	Sexual abuse (n = 719)	Harsh discipline (n = 719)
Not exposed	479	599	627	353
Slightly or not at all	1	0	1	38
A little	10	3	9	69
Moderately	18	10	13	85
Quite a bit	75	38	15	94
Extremely	136	69	54	80
Total exposed n(%)	240 (33.4%)	120 (16.7%)	92 (12.8%)	366 (50.9%)
**Duration** if exposed M(SD)	190 (166) months or 15.8 (13.8) years	114 (90) months or 9.5 (7.5) years	60 (57) months or 5 (4.75) years	108 (69) months or 9 (5.75) years
**Environmental stressors** Range 0–8 (total n = 719)	1 stressor	2 stressors	3 stressors	4 or more stressors
PwMS exposed to each count of stressors (n = 255 with no exposure)	165	113	115	101

### Fatigue

The base model block of predictors contributed to a significant overall two-part model estimating the likelihood and magnitude of having fatigue (logistic regression pseudo R^2^ = 0.074, p<0.04; OLS regression R^2^ = 0.087, p<0.001; model AIC = 4232) (Table 4). Childhood emotional stressors in model 2 contributed a significant amount of variance over and above the base model (logistic regression pseudo R^2^ = 0.096, p<0.05; OLS regression R^2^ = 0.111, p<0.001; AIC = 4219, LR p<0.001). Childhood physical stressors (model 3) significantly contributed over prior nested models (logistic regression pseudo R^2^ = 0.135, p<0.03; OLS regression R^2^ = 0.142, p<0.001; AIC = 4214, LR p<0.01). Childhood environmental stressors in model 4 did not significantly contribute as a block over the nested models and greatly reduced model fit (AIC = 4982; LR p = 1.0), and thus was removed from the final analytic model shown in Table 3.4. However, model 4 was a significant model overall (logistic regression pseudo R^2^ = 0.14, p<0.03; OLS regression R^2^ = 0.149, p<0.001), and before being removed, childhood environmental stress count was individually significantly associated with magnitude of fatigue (b = 0.42, p = <0.05). Therefore, only childhood emotional and physical stressors were included in the best fitting, final analytic model; yet environmental stressors do appear to additionally contribute to MS fatigue outside of the best fitting model. In addition to the blocks or clusters of similar predictors, harsh discipline duration was also independently significantly associated with the magnitude of fatigue (b = 0.02, p<0.03).

**Table 4:** Fatigue assessment using two-part regression modeling.

	First part—logistic regression (n = 606) Any fatigue (binary)					Second part—OLS regression (n = 582) Magnitude of fatigue				Overall model stats		
	OR	SE	95% CI	p	Pseudo R^2^	b	SE	95% CI	p	R^2^	AIC	LR tests
**Base covariates**				**<0.04**	0.0739				**<0.0001**	0.086	4232	base
**Age**	1.001	0.02	0.96–1.04	0.97		-0.07	0.03	-0.12 - -0.01	**<0.02**			
**Gender** (ref. female)												
Male	0.65	0.35	0.23–1.88	0.43		-0.75	0.92	-2.54–1.04	0.41			
**Education** (ref. ≤HS)												
Bachelor’s degree	0.13	0.14	0.02–1.03	0.053		-2.85	0.82	-4.45 - -1.25	**<0.001**			
Master’s degree or above	0.15	0.16	0.02–1.22	0.08		-4.60	0.81	-6.18 - -3.03	**<0.001**			
**DMT** (ref. No therapy)												
First line	0.64	0.44	0.17–2.44	0.51		-1.40	0.97	-3.30–0.50	0.15			
Second line	2.09	1.65	0.44–9.84	0.35		0.05	0.97	-1.86–1.95	0.96			
**MS Phenotype** (ref. RRMS)												
SPMS	1.29	1.05	0.26–6.39	0.76		2.52	0.99	0.59–4.46	**0.01**			
**Emotional stressors**				**0.02**	0.0964				**<0.0001**	0.1106	4219	**0.0004**
Emotional abuse severity	0.96	0.21	0.62–1.48	0.84		0.42	0.23	-0.02–0.87	0.06			
Emotional abuse duration	1.01	0.01	1.00–1.03	0.20		-0.003	0.003	-0.01–0.004	0.46			
**Physical stressors**				**<0.03**	0.1353				**<0.0001**	0.1420	4214	0.004
Physical abuse severity	0.71	0.17	0.45–1.13	0.15		-0.43	0.33	-1.08–0.21	0.91			
Physical abuse duration	1.004	0.01	0.99–1.02	0.71		0.01	0.01	-0.01–0.03	0.41			
Sexual abuse severity	1.04	0.93	0.18–5.95	0.96		0.60	0.33	-0.06–1.24	0.07			
Sexual abuse duration	1.17	0.42	0.57–2.38	0.67		0.002	0.02	-0.03–0.04	0.93			
Harsh discipline severity	1.38	0.33	0.86–2.19	0.18		0.07	0.28	-0.48–0.61	0.81			
Harsh discipline duration	0.99	0.005	0.98–1.00	0.07		0.02	0.01	0.002–0.03	**0.02**			

*The following categories of variables were dropped due to collinearity: 1) Transgender, non-binary, gender non-conforming, 2) Associates degree or some college, 3) PPMS, 4) PRMS, 5) Unsure

### Pain interference

The base model block of predictors contributed to a significant overall two-part model estimating the likelihood and magnitude of having pain interference (logistic regression pseudo R^2^ = 0.223, p<0.001; OLS regression R^2^ = 0.19, p<0.001; model AIC = 3784) (Table 5). Childhood emotional stressors in model 2 contributed a significant amount of variance over and above the base model (logistic regression pseudo R^2^ = 0.2379, p<0.001; OLS regression R^2^ = 0.204, p<0.001; AIC = 3770, LR p<0.01). Childhood physical stressors in model 3 significantly contributed over prior nested models (logistic regression pseudo R^2^ = 0.247, p<0.001; OLS regression R^2^ = 0.237, p<0.001; AIC = 3766, LR p<0.01). In model 4, childhood environmental stressors did not significantly contribute as a block over the nested models and reduced model fit (AIC = 3768; LR p = 0.4), and thus was removed from the final analytic model shown in Table 5. Therefore, model 3 including emotional and physical childhood stressors, was the best fitting model. For each 1-unit increase in emotional abuse severity rating there was nearly a 16% increase in the odds of experiencing any pain interference, yet this was slightly over the significance threshold (OR = 1.16, p = 0.057). Harsh discipline was significantly associated with the magnitude of pain interference (b = 0.014, p = 0.019).

**Table 5:** Pain interference assessment using two-part regression modeling.

	First part—logistic regression (n = 707) Any pain interference (binary)					Second part—OLS regression (n = 464) Magnitude of pain interference				Overall model stats		
	OR	SE	95% CI	p	Pseudo R^2^	b	SE	95% CI	p	R^2^	AIC	LR test
**Base covariates**				**<0.0001**	0.2231				**<0.0001**	0.1881	3784	Base
**Age**	1.02	0.01	1.01–1.04	**<0.01**		-0.10	0.03	-0.16 - -0.05	**<0.001**			
**Gender** (ref. female)												
Male	0.64	0.17	0.38–1.07	0.09		0.85	0.94	-0.99–2.69	0.37			
Transgender, non-binary, gender non-conforming, other	1.30	0.93	0.32–5.27	0.72		-1.40	1.97	-5.26–2.45	0.48			
**Education** (ref. ≤HS)												
Associates degree or some college	0.16	0.13	0.03–0.76	**0.02**		-1.32	1.26	-3.79–1.14	0.29			
Bachelor’s degree	0.09	0.07	0.02–0.42	**<0.01**		-4.40	1.23	-6.80 - -2.00	**<0.001**			
Master’s degree or above	0.09	0.07	0.02–0.40	**<0.01**		-4.78	1.23	-7.19 - -2.37	**<0.0001**			
**MS Phenotype** (ref. RRMS)												
PPMS	1.23	0.58	0.48–3.11	0.67		-0.24	1.41	-3.01–2.52	0.86			
SPMS	1.40	0.47	0.72–2.70	0.32		2.39	0.93	0.56–4.21	**0.01**			
PRMS	1.10	1.02	0.18–6.81	0.92		6.11	2.42	1.36–10.86	**0.01**			
Unsure	1.04	0.57	0.36–3.03	0.94		2.84	1.74	-0.57–6.26	0.10			
**DMT** (ref. No therapy)												
First line	1.35	0.38	0.77–2.36	0.29		-0.51	0.93	-2.33–1.31	0.59			
Second line	2.13	0.62	1.20–3.78	<0.01		-1.35	0.89	-3.09–0.39	0.13			
**Pain med count**	2.45	0.24	2.03–2.97	<0.001		1.38	0.24	0.92–1.84	**<0.001**			
**Emotional stressors**				**<0.0001**	0.2379				**<0.0001**	0.2037	3770	**0.0002**
Emotional abuse severity	1.16	0.09	1.00–1.34	0.057		-0.06	0.20	-0.46–0.33	0.75			
Emotional abuse duration	1.00	0.001	1.00–1.002	0.52		0.003	0.003	-0.003–0.01	0.35			
**Physical stressors**				**<0.0001**	0.2468				**<0.0001**	0.2372	3766	**0.006**
Physical abuse severity	1.02	0.11	0.83–1.26	0.86		0.18	0.28	-0.36–0.72	0.52			
Physical abuse duration	1.00	0.003	0.99–1.01	0.76		-0.01	0.01	-0.03–0.01	0.25			
Sexual abuse severity	1.05	0.11	0.85–1.29	0.66		0.33	0.28	-0.23–0.88	0.25			
Sexual abuse duration	1.002	0.01	0.99–1.01	0.73		0.02	0.02	-0.02–0.05	0.34			
Harsh discipline severity	1.001	0.09	0.85–1.19	0.99		0.05	0.25	-0.44–0.54	0.85			
Harsh discipline duration	1.004	0.002	1.00–1.01	0.068		0.01	0.01	0.002–0.03	**0.019**			

### Psychiatric morbidity

The base model block of predictors significantly contributed to estimating psychiatric morbidity (Pseudo R^2^ = 0.062, p<0.001; model AIC = 2579) (Table 6). Childhood emotional stressor characteristics in model 2 contributed a significant amount of variance over the base model (Pseudo R^2^ = 0.085, p<0.001; AIC = 2519, LR p<0.0001). Childhood physical stressors in model 3 contributed significantly more information over the prior nested models (Pseudo R^2^ = 0.092, p<0.001, AIC = 2513, LR p<0.01). Childhood environmental stressors in model 4 also contributed significantly over prior nested models (Pseudo R^2^ = 0.094, p<0.001, AIC = 2508, LR p<0.01). Therefore, all childhood stressors contributed significantly and remained in the final analytic model.

**Table 6:** Psychiatric morbidity assessment using Poisson regression (n = 711).

					Overall model statistics		
	IRR	SE	95% CI	p	Pseudo R^2^	AIC	LR test
**Base covariates**				**<0.0001**	0.0616	2579	base
**Age**	0.988	0.002	0.98–0.99	**<0.001**			
**Gender** (ref. female)							
Male	0.84	0.07	0.71–1.00	**<0.05**			
Transgender, non-binary, gender non- conforming	1.04	0.16	0.76–1.41	0.82			
**Education** (ref. ≤HS)							
Associates degree or some college	0.88	0.10	0.70–1.09	0.24			
Bachelor’s degree	0.80	0.09	0.64–0.99	**<0.05**			
Master’s degree or above	0.79	0.09	0.64–0.98	**<0.05**			
**MS Phenotype** (ref. RRMS)							
PPMS	1.15	0.15	0.90–1.48	0.27			
SPMS	1.05	0.09	0.88–1.24	0.62			
PRMS	1.23	0.25	0.83–1.82	0.31			
Unsure	1.17	0.17	0.88–1.54	0.29			
**Pain med count**	1.15	0.02	1.11–1.20	**<0.001**			
**Emotional stressors**				**<0.0001**	0.0853	2519	**<0.0001**
Emotional abuse severity	1.06	0.02	1.02–1.10	**0.002**			
Emotional abuse duration	1.00	0.0003	1.00–1.001	0.57			
**Physical stressors**				**<0.0001**	0.0916	2513	**0.0086**
Physical abuse severity	0.97	0.02	0.92–1.01	0.17			
Physical abuse duration	1.00	0.001	1.00–1.002	0.80			
Sexual abuse severity	1.03	0.02	0.99–1.08	0.17			
Sexual abuse duration	1.00	0.001	1.00–1.002	0.99			
Harsh discipline severity	1.01	0.02	0.97–1.06	0.61			
Harsh discipline duration	1.00	0.001	1.00–1.002	0.09			
**Environmental stressor**				**<0.0001**	0.0942	2508	**0.0075**
Environmental stressor count	1.05	0.02	1.01–1.09	**0.007**			

Regarding key individual predictors in the final model, for each additional childhood environmental stressor experienced, participants had 4.8% increased risk of accumulating an additional mental health symptom, diagnosis, or event (IRR = 1.05, p<0.01). For example, those who experienced eight environmental stressors during childhood had 40% more risk of psychiatric morbidity compared to those who had no exposure to environmental stressors. Similarly, for each 1-unit increase in emotional abuse severity rating, the risk of accumulating psychiatric morbidity increased by 6% (IRR = 1.06, p<0.01). For example, those who rated emotional abuse as extremely severe have 30% more risk of accumulating a new mental health symptom, diagnosis, or event, compared to those who were not exposed to emotional abuse.

## Discussion

This work builds upon research that suggests that childhood adversity influences symptoms commonly experienced by people with MS including pain [32], fatigue [19], and mental health [15, 33]. However, to our knowledge, this is the first study to assess clusters of similar childhood stressor characteristics including severity and duration, to assess their joint contributions for estimating key chronic MS symptoms. Results show that childhood emotional and physical stressors significantly relate to MS fatigue, pain interference and psychiatric morbidity, while childhood environmental stressors were additionally associated with psychiatric morbidity (Summary Table 7).

**Table 7:** Summary of stressor associations across all outcomes.

	Predictor blocks included in final model	Additional significant individual stressor contributions to MS outcomes
**Fatigue**	• Emotional and physical	• Harsh discipline duration
**Pain Interference**	• Emotional and physical	• Harsh discipline duration
**Psychiatric morbidity**	• Emotional, physical, and environmental	• Emotional abuse severity • Environmental stressor count

The stressor predictor blocks were ordered to first capture stressors that happen *to* a child (i.e., emotional, physical), then stressors that happen *around* a child (i.e., environmental). However, this ordering could have underestimated the impact of environmental stressors, especially in the two-part models. In the two-part models, adding one variable is treated as if two were added, because there are two beta coefficients and an additional degree of freedom (on which the likelihood ratio test depends). There is some evidence of this in the two-part fatigue analysis. The final environmental block did not contribute above and beyond the previous emotional and physical blocks when tested with the likelihood ratio test. However, the individual environmental count variable had a significant p-value (p = 0.048) in only the second part of the model for estimating fatigue magnitude. This suggests that had the environmental block been added earlier instead, it may have contributed significantly more and stayed in the most parsimonious models for more outcomes.

In addition to the blocks of predictors having joint significance, some predictors were also individually significant across outcomes (Table 7). Stressor severity and duration contributed differently across outcomes in this study, suggesting the potential for count-based analyses in current or future literature to not fully capture this nuanced information; this could lead to a type II error of underestimated or null results while there may truly be a relationship. In our study, emotional abuse severity significantly related to psychiatric morbidity; and nearly reached significance with fatigue magnitude (p = 0.06) and reporting any pain interference (p = 0.057). Harsh discipline duration was significantly associated with both the magnitude of fatigue and pain interference. Environmental stressors significantly related to fatigue magnitude and psychiatric morbidity. Sexual abuse severity was nearing significance with fatigue magnitude, however, lower prevalence and/or reporting may have impacted the ability to determine significance. Yet, the rates of abuse reported in this study are very consistent with the emotional (33.4% vs. 34.4%), physical (16.7% vs 17.9%), and sexual abuse (12.8% vs. 11.6%) rates found through national surveillance work [2].

It is possible that psychiatric morbidity could have partially mediated the observed relationship between childhood stress and fatigue [19]. Fatigue is ambiguous, making it challenging to disentangle what portion is strictly from MS versus symptoms of depression, for example. Cognitive and emotional responses pathways could lead to higher likelihood of perception of pain, fatigue, and mental health struggles.

Several demographic characteristics also made contributions. Not surprisingly, higher education was consistently related to better results across all outcomes. This study sample was highly educated, however, this was not unexpected as large MS samples have also shown to have a high percentage of participants with degrees [51]. Most (71%) participants obtained at least a bachelor’s degree, suggesting that the relationships found could be even stronger for other samples who may have experienced additional adversity and barriers to education. This same phenomenon has been observed throughout many other health outcomes and for overall health [52, 53], again highlighting the need to invest in programs that address barriers and facilitate child, adolescent, and adult educational success to help promote health over the lifespan [54].

The risk for experiencing *any* pain interference increased with age. Conversely, psychiatric morbidity and the *magnitude* of fatigue and pain interference all decreased with age. This may represent better disease management, better coping skills, and perhaps engaging in mental health support happening over time since their diagnosis. Yet it may also represent fewer straining activities as PwMS may cut back on job or family responsibilities over time.

### Implications

These study findings expand upon the many known detrimental health outcomes stemming from adverse childhood experiences to include negative impacts on three chronic debilitating symptoms in MS. Yet, this area is still understudied and warrants future research, which should include critical MS confounders and consider how social, educational, racial, and ethnic factors can best be assessed in the context of stressors and MS. Results should be interpreted with caution since the sample may not represent all PwMS, and future research should enhance generalizability.

This work supports the current movement to integrate trauma informed care and stressor screening into clinical practice, especially for PwMS. Screening could enable neurologists to be more informed about patient backgrounds and risk factors that predict disease trajectories, thereby contributing to personalized approaches to MS care. For example, if screening were integrated into the process while establishing care with a new patient, neurologists could discern that their patient with high childhood stressors was at higher risk of worsening anxiety over the year following an MS diagnosis [15], and refer them to mental health support to mitigate psychiatric morbidity. Improved coping skills may also facilitate smoking cessation and prompt health behavior change (e.g., improve sleep, diet, physical activity) which may help improve MS disease burden [55, 56]. In addition, there is also some evidence to suggest that engaging PwMS with mental health support may help alleviate symptoms of fatigue, pain, relapses, possibly leading to less disability, increased quality of life and treatment adherence [18, 19, 32].

A potential clinical implication from this work is the generation of data that could inform more personalized treatment options for MS symptoms, based on stressor exposure. Evidence suggests that some PwMS respond similarly to pharmacological therapy or a placebo in the treatment of fatigue [57], while Braley et al., (2023) found that Cognitive Behavioral Therapy (CBT) yielded similar results to pharmacological therapy (modafinil) [58]. CBT has also shown to be helpful for mental health and DMT adherence when PwMS struggle with the decision to stop treatment against medical advice [59]. Future work should help determine if those with childhood stressors and fatigue respond better to non-pharmacologic options like CBT, which may even be increasingly beneficial.

Screening could be inclusive of adult stressors, as both child and adult stressors over the lifespan have recently been associated with MS disability [60]. While patients often share current stressors with their neurologists, there is evidence to suggest that formal screening is not a regular part of practice despite the American Academy of Neurology position supporting screening for past and current trauma [61–63]. There is also evidence that healthcare providers are uncomfortable screening because they are unsure of what steps to take next [64]. Training and published guidelines help guide providers through these conversations with patients. Resources like Aces Aware (acesaware.org) and the Trauma Informed Care Implementation Center (https://www.traumainformedcare.chcs.org) can help healthcare providers learn about, train for, and implement trauma informed care. Lastly, policy changes in some states have facilitated trauma screening by higher billing reimbursement rates of around $30 [65], while some states reimbursement rates are less than $3 [66]. Widespread screening reimbursement could help improve screening practices in various healthcare settings and states.

### Limitations

The results, and thus conclusions, should be interpreted with caution as this study was conducted online due to COVID-19 research restrictions so the sample composition may not be generalizable to all PwMS. There is likely a selection bias due to the low participant response rate (approximately 0.01%) in comparison to the NMSS listserv size (80,000 PwMS) and relatively high education levels. Thus, this sample of respondents may not be representative of those without the technological, physical, or cognitive ability to participate in the survey, those who were not as impacted by traumatic stressors, who were not interested in the research opportunity, or those with lower education levels. Despite the response rate, and evidence that White women are more likely to respond to online surveys which could lead to overrepresentation, this sample generally aligns with the US MS research population, and has some strengths regarding internal and external validity [67, 68]. For example, the MS sample size is larger than all other known studies in this research area, and includes the largest geographical range which is especially important for MS as incidence and prevalence rates vary by location.

An inherent limitation with any self-administered questionnaire that includes items about current or past sensitive topics is the potential for recall and social desirability bias. However, the STRAIN has been shown to be reliable over time, not induce negative mood, or have social desirability bias (33). Additionally, the rates of abuse in this study are very consistent with national rates (2). However, this study did not include emotional neglect which could lead to underestimated results. The cross-sectional design limits the ability to assess temporal ordering or causation. However, many other studies have found meaningful results using these methods and given the small but growing state of the science in this area, this design provides a foundation for future prospective work.

## Conclusion

These findings support the hypothesized relationships among childhood adversity and fatigue, pain interference, and psychiatric morbidity in PwMS. Groups of similar stressors, emotional, physical, or environmental, had differing influences across the outcomes, suggesting multiple pathways from stress to MS symptoms. More studies are needed to add to the scarce literature in this area, replicate results, and expand to unexplored clinical features such as sleep, cognitive function, and response to treatments to better support trauma informed precision medicine.
